# Concavo-convex oblique anastomosis technique for jejuno ileal atresia

**DOI:** 10.4103/0971-9261.59603

**Published:** 2009

**Authors:** Zaheer Hasan, A.N. Gangopadhyay, Punit Srivastava, Mohammad Akhtar Hussain

**Affiliations:** Department of Pediatric Surgery, IMS, BHU, Varanasi, India

**Keywords:** Concavo-convex oblique anastomosis, end to back anastomosis, jejuno-ileal atresia

## Abstract

**Aim:**

To evaluate the role of end-to-end oblique bowel anastomosis in bowel atresia.

**Methods:**

End-to-end oblique anastomosis was done in 25 neonates of bowel atresia and the results were compared with traditional method of end to back anastomosis in 25 cases.

**Results:**

We found less mortality and morbidity (5%) in our technique as compared to end to back technique (20%).

**Conclusions:**

We recommend this technique, as anastomosis is wide with less angulations, flow of effluent is linear, and there is less force exerted over post-anastomotic side wall.

## INTRODUCTION

The incidence of jejuno-ileal atresia varies from 1 in 330 to 1 in 1500 live births. In cases of small bowel atresia, dilated bowel segment usually involves large portion of proximal intestine, so at what ever point resection is performed, the discrepancy in diameter between proximal and distal segment does not change; thus performing anastomosis is a difficult task. Although, there are many anastomotic techniques had been developed, they are prone to develop stricture at anastomotic site in long term.

## MATERIALS AND METHODS

The present study has been conducted from 2002 to 2008. Our technique of performing anastomosis was compared with that of conventional end to back anastomosis. We selected 25 neonates randomly (test group) on whom (end to end concavo-convex) anastomosis was done and results were compared with that of 25 neonates (control group) in which traditional end to back anastomosis were done. Both the groups were age and weight matched. We excluded type IIIb and IV atresia and patients of major congenital anomalies. Entire surgery was performed by the same surgeon and in the random fashion. Neonates presented within 24 h to 4 days of life. Out of them, 10 patients were less than 24 h and 15 were more than 24 h in both groups. Out of 50 patients, 30 were males and 20 were females. Among 50 patients, 16 were under 2 kg, 26 were between 2 to 2.5 kg, and 8 were more than 2.5 kg. Jejunoileal atresia was diagnosed on the basis of history, clinical examination, and x-ray of abdomen.

**Figure 1:**
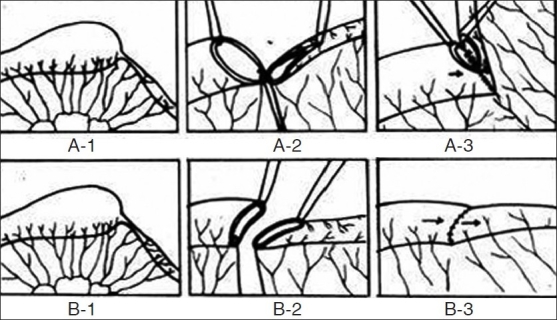
(A1-3) Diagram showing conventional anastomotic technique showing force of propulsion hitting the side wall and functional anastomotic area is narrow. (B1-3) Diagram showing our technique of anastomosis showing force of propulsion is linear and anastomotic area is wide

Exploration of abdomen was done by right transverse supra-umbilical incision. Proximal dilated and distal constricted bowel segments were identified. Proximal distended segment was deflated and 5–10 cm of distended bowel was resected. Margin was cut obliquely starting from antimesenteric border to mesenteric border making cut margin concave so that the 1 cm difference created between antimesenteric and mesenteric border simultaneously preserving mesenteric vascular supply. Distal atretic bowel wall was cut obliquely making cut margin look convex similarly creating 1 cm difference between antimesenteric and mesenteric border [Figure 1]. Anastomosis was performed in single layer interrupted by 5-0 vicryl sutures. We first approximate mesentery of adjacent bowel. Inverting sutures were taken in interrupted manner. Two or three interrupted stitches were taken commencing from mesenteric border and anastomosis was progressed toward antimesenteric border from opposite direction, thereby completing anastomosis. Last two to three anterior stitches were taken full thickness Lembert, which gives better serosa to serosa approximation. Finally, mesenteric defect was closed.

Post-operatively, patients were given IV fluids, antibiotics, cholestyramine, and kept nil by mouth and continuous nasogastric aspiration by infant feeding tube. Patients usually start passing meconium from 3rd or 4th day onward. Patients were given nasogastric feed on 5 to 6 day post-operatively routinely and full oral feed 8th to 10th day depending upon tolerability. Average hospital stay was 10 days. All the patients were regularly followed up in OPD.

## RESULTS

Out of 50 neonates, 25 underwent oblique anastomosis (our technique) and 25 were end to back anastomosis (conventional).Only, one patient in our technique died due to septicemia and low birth weight mortality rate was 1 in 25 (4%). Five patients died out of 25 in end to back anastomosis, mortality rate was 5 in 25 (20%) due to leak at the site of anastomosis. In our technique, oral feeds were started on 5.56 ± 0.76 whereas in end to back anastomosis it was on 9.76 ± 0.72 day. There is a significant difference between the day of starting feeding between the groups (t value= 19.9025; df = 48; p-value= 0.000). In end to back anastomotic technique, 6 patients out of 25 (24%) developed features of subacute intestinal obstruction characterized by abdominal distention, vomiting, and excess nasogastric aspirate but had resolved upon conservative management. However, in our technique none of the patients developed features of intestinal obstruction.

## DISCUSSION

Many anastomotic techniques have been devised for intestinal atresia. The procedures can be classified into two types: (1) widening of caliber of the diminutive distal bowel and (2) reducing the caliber of the large proximal bowel. End to back, end to side, and end to end oblique anastomosis that is described belong to first type, and tapering enteroplasty[1] followed by end to end anastomosis into the second type. In 1894, Wanitschek[2] attempted first resection anastomosis for the intestinal atresia unsuccessfully. End-to-back anastomosis[3,4] shows neither technical problems nor post-operative anastomotic functional obstruction if the caliber ratio between the proximal and distal atretic bowels is not large. However, as the caliber ratio increases longitudinal axis deviation between proximal and distal bowels gradually becomes close to 90°, resembling end to side anastomosis, which easily results in functional obstruction. It seems to be very difficult to perform functional end-to-back anastomosis in cases where caliber ratio is more than 4. Gambee's[5] single layer anastomosis, which was originally performed in an end to end anastomotic fashion, has no anastomotic axial deviation, but the caliber ratio must be limited to four values to perform this procedure safely.[6] Halsted's aseptic anastomosis[7] also resulted in no anastomosis axial deviation and is reported to be possible in cases with a caliber ratio of upto five values although there must be limit for its indication.[8]

Tapering jejunoplasty[9] of the proximal dilated segment followed by construction of end to end jejunostomy has been reported to be useful[10–12] for large caliber difference. However, this method has disadvantage of bowel dismotility and axial deviation. Another technique described by Patil *et al* in which linear anastomosis was done excising a circular disc of proximal dilated bowel and anastomosed to narrowed distal bowel in single layer, but in this case there was luminal disparity in proximal and distal segment causing leakage at anastomotic site and increased morbidity and mortality rate.[13]

In our technique, we cut proximal bowel in concave fashion after 5–10 cm bowel resection and distal bowel in convex fashion and performed anastomosis in interrupted manner with vicryl 5-0 (round body). The resultant anastomosis is oblique and patulous one without bowel angulations. Furthermore, flow of intestinal content is linear one; hence there is less shearing force at anastomotic site. Also, this technique does not require Cheatle's maneuver.

Since our anastomosis is oblique and patulous, flow of effluent is streamline and linear and there is less pressure over the side wall, there is less chance of anastomotic leakage. In fact, there was no leak in our technique as demonstrated by post-operative dye study.

## CONCLUSION

This technique of end to end oblique anastomosis results in wide and early functioning anastomosis. The mortality and morbidity rate is reduced. Hospital stay is also reduced. Total parenteral nutrition is not required. We believed that improved results are due to fewer angulations, better alignment of adjacent bowel loop, and wider functional anastomotic area resulting in linear flow of effluent. So, we recommend this method of bowel anastomosis in cases of jejunoileal atresia as a technical advancement.
